# Characteristics and Treatment of *Gordonia* spp. Bacteremia, France

**DOI:** 10.3201/eid2905.221901

**Published:** 2023-05

**Authors:** Alia Barthel, Axel Ursenbach, Charlotte Kaeuffer, Christelle Koebel, Alain Gravet, Dominique De Briel, Jacqueline Dubois, Elina Haerrel, Estelle Rougier, Victor Gerber

**Affiliations:** Hôpitaux universitaires de Strasbourg, Strasbourg, France (A. Barthel, A. Ursenbach, C. Koebel, E. Rougier);; Groupement Hospitalier de Mulhouse et Sud-Alsace, Mulhouse, France (C. Kaeuffer, A. Gravet);; Hôpitaux Civils de Colmar, Colmar, France (D. de Briel, J. Dubois, E. Haerrel, V. Gerber)

**Keywords:** *Gordonia*, bacteremia, bacteria, Gram-positive, nosocomial infections, antimicrobial resistance, opportunistic infections, catheter-related infection, France

## Abstract

Systemic *Gordonia* spp. infections are rare and occur mostly among immunocompromised patients. We analyzed 10 cases of *Gordonia* bacteremia diagnosed in 3 tertiary care centers in France to assess risk factors, treatment, and clinical outcomes. Most patients were cured within 10 days by using β-lactam antimicrobial therapy and removing central catheters.

*Gordonia* is a slow-growing, gram-positive aerobic bacillus of the order *Mycobacteriales*. More than 40 species of this ubiquitous environmental bacterium have been described, few of which seem to infect humans (*1*). Some localized, postoperative, or traumatic infections have been described (*2*,*3*). Among immunocompromised patients, systemic bloodstream infections, although relatively uncommon, can be caused by contaminated biofilm adherent on endovascular devices (*4*,*5*). However, cases of secondary infection are more uncommon. 

*Gordonia* bacteremia is not well understood because of its rarity; in addition, blood cultures may not be positive until >4 days after infection (*6*). The use of 16S ribosomal RNA gene sequencing has improved *Gordonia* identification rates over results from other, often insufficient, standard laboratory tests, which sometimes led to misidentification (*7*). Because no standardized recommendations for treatment exist, reported cases have been managed heterogeneously (*4*,*5*); a single treatment regimen, a 4–6-week course of an aminoglycoside combined with a carbapenem or fluoroquinolone, developed based on a 5-case series study and a literature review, has been proposed (*7*). Because some cases of severe infections have been described (*8*), to clarify risk factors and management options, we initiated this investigation describing patient characteristics to examine treatment outcomes from cases of *Gordonia* bacteremia. Our study was approved by the Ethics Committee of Medicine, Odontology and Pharmacy Faculties and Hospitals of the University Hospital of Strasbourg (project no. CE-2022-112) and declared to the Commission Nationale Informatique et Libertés. 

## The Study 

We retrospectively reviewed medical records from 2 tertiary care centers and 1 university hospital in northeast France. Our main purpose was to assess how *Gordonia* bacteremia cases had been managed. Our secondary purpose was to identify risk factors associated with infection. We requested information from the laboratory database of each center to collect all blood cultures testing positive for *Gordonia* spp. during January 1, 2010–May 31, 2021. We included patients with blood cultures positive for *Gordonia* sampled while hospitalized in one of the centers during the study period. We sought informed consent by mailing information about the study to patients or the parents of pediatric patients. We excluded persons opposed to participation and adults under legal guardianship or conservatorship. From medical files, we collected data on underlying diseases and immunodeficiency; presence and type of long-lasting intravascular device use, if any; clinical characteristics; antimicrobial susceptibility; treatments; and outcomes. We considered outcomes favorable when complete clinical cure was achieved, as determined by normalization of blood tests and repeated negative blood cultures. We sought relapses that occurred through May 31, 2022, or death of the patient.

We included 10 patients (median 49 years of age), among whom 7 had immunodeficiency and 6 had immunosuppression factors, most commonly severe malnutrition or long-term corticosteroid therapy (Tables 1, 2). Fever was the most common clinical sign; no secondary infection site was reported among case-patients. Healthcare providers considered *Gordonia* a contaminant in medical records from 5 cases; 4 patients had no central venous catheter, 3 were immunocompetent, and 1 had controlled HIV infection. We identified subspecies *G. sputi*, *G. bronchialis*, *and G. aichiensis*. Despite 16S rRNA gene sequencing, among 3 case-patients bacterium identification was limited to the genus level (i.e., *Gordonia* spp.). Nine patients were empirically prescribed antimicrobial therapy, mostly with third-generation cephalosporins and β-lactam/β-lactamase inhibitor combinations, for a median duration of 15 days. All *Gordonia* spp. were susceptible to treatment in all cases. In the 3 cases in which a long-term catheter had been removed, apyrexia without relapse was obtained within a few days of device removal; median treatment period was shorter, 13 days (range 0–15 days), for those patients who had their catheters removed than for patents who had not had their catheters removed (15 days, range 7–32 days). One patient was cured, as assessed by clinical improvement and negative repeated blood sample, by device removal alone without antimicrobial treatment. Of 2 patients who experienced relapses, 1 recovered promptly after delayed catheter removal, and the other exhibited febrile relapses with no other obvious cause of fever (e.g., venous thrombosis), clinical signs of catheter infection, and repeated blood cultures positive for *Gordonia*. 

**Table 1:** Description of *Gordonia* bacteremia cases, 2010–2021, France*

Patient no.	Patient age/sex	Immune deficiency factors	CVC type	Clinical signs	Species (no. positive samples)	AMR	Treatment	Time, d	CVC removed
Favorable outcome†									
1	36 y/M	HIV, nephrotic syndrome	HD	CVC inflammation	*Gordonia* sp. (1)	None	None	0	Yes
2	3 y/M	Hemopathy, corticosteroid, methotrexate	Central port	Fever	*G. bronchialis *(1)‡	None	CAZ, AMC	7	No
3	86 y/M	None	None	Fever, cough	*G. sputi* (1)‡	None	AMC, 3GC	10	NA
4	56 y/F	HIV	None	Fever	*G. sputi* (1) ‡	None	3GC	10	NA
5	67 y/F	Malnutrition, hemopathy, cancer	TCVC	Acute kidney failure	*Gordonia* sp. (1)	None	3GC, VAN	15	Yes
6	75 y/M	None	None	Fever, chills	*G. aichiensis* (1)‡	ERY, FA, FOF	PEN G, AMOX	15	NA
7	42 y/M	Cirrhosis splenectomy, corticosteroid	Central port	Fever	*G. sputi *(2)	PEN, SMX	TZP, VAN	15	No
8	2y/M	None	None	Knee arthritis	*Gordonia* sp. (1)‡	§	CFZ, AMK; Tr 2¶: cefaclor	5	NA
								30	
Relapse#									
9	3 mo/M	Nephrotic syndrome, low IgG	TCVC	Fever	*G. bronchialis* (1)	ERY, FA, FOF	AMOX; Tr 2¶: JM, polyvalent Ig	8	Yes
								5	
10	61 y/F	Immunosuppressive regimen, corticosteroid, malnutrition	Central port	Fever, angiocolitis, port inflammation	*G. sputi *(2)	§	CPFX, TZP; Tr 2¶: IMP, AMK, VAN; Tr 3¶: 3GC, GEN	15	No
								10	
								7	

*3GC, 3rd generation cephalosporin; AMC, amoxicillin-clavulanic acid; AMK, amikacin; AMOX, amoxicillin; AMR, antimicrobial resistance; CAZ, ceftazidime; CFZ, cefazolin; CPFX, ciprofloxacin; CVC, central venous catheter; ERY, erythromycin; FA, fusidic acid; FOF, fosfomycin; GEN, gentamicin; HD, hemodialysis; IMP, imipenem; JM, josamycin; NA, not applicable; PEN, penicillin; SMX, sulfamethoxazole; TCVC, tunneled CVC; TZP, piperacillin/tazobactam; VAN, vancomycin. †Favorable outcome required clinical and biologic cure and repeated negative blood sample (when available), without relapse until the end of the study period (May 31, 2022). ‡In medical record, *Gordonia* sp. was considered a contaminant with no clinical significance. §Antimicrobial susceptibility testing not performed. ¶Treatment regimens after relapse. #Relapses through May 31, 2022.

**Table 2:** Characteristics of patients with *Gordonia* bacteremia, 2010–2021, France*

Variables	No. (%)
Median age, y (range)	49 (0.25–86.0)
Sex, n = 10	
M	6 (60.0)
F	4 (40.0)
Immunodeficiency factors, n = 7	
Corticosteroids	3 (42.8)
Severe malnutrition	4 (57.1)
Hemodialysis	2 (28.6)
Hemopathy	2 (28.6)
Transplantation	2 (28.6)
HIV	2 (28.6)
Central venous catheter, n = 6	
Central port	3 (50.0)
Other central catheter	3 (50.0)
Nosocomial infection,* n = 10	
Y	3 (30.0)
N	7 (70.0)
Microbiological identification, n = 10	
* G. bronchialis*	2 (20.0)
* G. sputi*	4 (40.0)
* G. aichiensis*	1 (10.0)
Antimicrobial therapy, n = 9	
3rd/4th-generation cephalosporin	6 (66.6)
β-lactam with β-lactamase inhibitor	4 (44.4)
Glycopeptide	3 (33.3)
Penicillin	2 (22.2)
Aminoside	2 (22.2)
Carbapenem	1 (11.1)
Fluoroquinolone	1 (11.1)
Median treatment duration, d (range)	15 (0–35)
CVC ablation, n = 6	3 (50.0)
Outcome, n = 10	
Favorable outcome†	8 (80.0)
Relapse‡	2 (20.0)

*Clinical signs of infection starting >48 h after hospitalization †Favorable outcome required clinical and biologic cure and repeated negative blood sample, without relapse through the end of the study period (May 31, 2022). ‡Relapse without cure through May 31, 2022.

In this study, based on clinical information from an extensive series of *Gordonia* bacteremia infections among patients from several hospitals, we analyzed risk factors regardless of patient immune status. We found the main risk factors were immunosuppression and indwelling catheters of any type, consistent with findings from previous reviews (*4*,*5*,*7*,*9*). The preferred treatment, with third-generation cephalosporins or β-lactam/β-lactamase inhibitor combinations for 1–2 weeks, was of shorter duration than in most previous cases and did not match the proposed treatment described earlier (*4*,*7*). However, the antimicrobial drugs used were effective against all *Gordonia* species. Among 8 case-patients with favorable outcomes, only 2 received an aminoglycoside, suggesting it might be reserved for use in more severe cases. The strains’ susceptibility to many antimicrobials, except for lesser susceptibility to sulfamethoxazole in some other studies, agrees with data published elsewhere (*4*,*7*,*10*,*11*). Effects after removing the central venous catheter were inconsistent but removal generally resulted in favorable outcomes. Positive effects from catheter removal has also been reported in other cases and reviews (*7*,*9*,*12*). 

Our retrospective analysis of case management reflects difficulties related to late identification and uncertainty about the truly infectious nature of *Gordonia* spp. bacteria. Differences exist in the literature about whether a single positive blood culture should be considered contamination or actual infection. Some subspecies, such as *G. terrae* or *G. sputi*, seem more likely to be responsible for bacteremias; other species, such as *G. bronchialis*, are rarely represented (*4*,*5*). In this study, *G. terrae* was present in 4 patients, but *G. bronchialis* was found in at least 2 cases and was strongly considered a probable cause of true infection. 

In reported cases in which *Gordonia* was considered a contaminant, empiric antimicrobial therapy was stopped after the few days required to achieve negative blood culture results, after which clinical signs regressed and repeated blood samples were negative in most cases (*9*,*13*). Whatever the significance of this microbiological result, whether specific *Gordonia* spp. are agents of infection or contaminants, short-term treatment may suffice in the absence of a central catheter or if subsequent blood samples are consistently negative, as described elsewhere (*13*).

One limitation of our retrospective study was that data were collected over >11 years, during which time variations in risk level and management strategies may have altered the experience of the small number (n = 10) of included case-patients. In addition, including cases in which *Gordonia* spp. were considered potential contaminates rather than causes of infection may have positively biased treatment outcomes. No cultures were taken from catheters to confirm their role in bacteremia, and antimicrobial susceptibility was not tested in all cases. Thus, some resistant strains might exist that have not yet been identified. 

## Conclusions

In the absence of consensus recommendations, standardizing management of bacteremias caused by *Gordonia* spp. should be pursued. In cases with positive blood cultures, *Gordonia* should be considered infective in immunosuppressed case-patients, especially when a central venous catheter is present. β-lactam monotherapy can be empirically prescribed with aminoglycoside added in severe cases. After any central venous catheter has been removed and clinical signs improve, discontinuing antimicrobial drugs after 1–2 weeks should be considered. However, further studies are needed to clarify treatment duration and make more precise recommendations about managing a central catheter, especially when it is not removable. Studies on the effectiveness of antimicrobial lock therapy would help, possibly combined with lengthened systemic antimicrobial therapy. Given the sometimes serious nature of infection, especially among immunocompromised patients, developing treatment strategies and increasing awareness among clinicians of *Gordonia*-caused bacteremias would be of public health benefit. 
